# Letter To the Editor: ‘Comment J’ai Vaincu la Douler et l’Inflammation Chronique Par l’Alimentation’ by J Lagacè

**Published:** 2013-01-04

**Authors:** G Scarpati, M Pintore

**Affiliations:** 1 Department of Medicine, University of Salerno; 2 Department of Pain Medicine, Oliveto Citra Hospital

“Stubbing pain in the hands, finger joints blocked, severe pain in the back and knees, insomnia, depression. The life of Dr. Jacqueline Lagacé, like that of millions of people suffering from arthritis and osteoarthritis, is an ordeal, until the day he encounters in the hypotoxic diet Dr. Seignalet and decided to try it” This is the introduction to the book “ Comment j’ai vaincu la douleur et l’inflammation chronique par l’alimentation” of Jaqueline Lagacé, Canadian physician specializing in virology and immunology. In this book Dr. Lagacè explains the basics of the Seignalet nutrition who believes that this system has a preventive or beneficial in many diseases. Seignalet advocates a return to an ancestral type of nutrition, his scheme is based on a primarily qualitative approach of diet, he dismisses the food they considered potentially harmful to the human organism (foods cooked at high temperature and also, among others, wheat and dairy products, organic foods and favors). This nutritional approach is variously called by the author ancestral diet, diet or feeding hypotoxic original type. The mechanisms of action proposed by the author to explain the pathogenesis associated with certain foods and the effectiveness of their removal are not scientifically proven. The sample used is not sufficient for a reliable statistical approach that includes: study on a large number of patients with double blind method and rate quantified as positive, negative and with a controlled follow-up. The scheme Seignalet currently, despite the efforts required by its implementation and very reserved attitude of some of the scientific community about its effectiveness.

Seignalet Jean (1936–2003), pioneer of renal transplantation in Languedoc-Roussillon was oriented nutrition through his research in immunology. He developed theories about the relationship between diet and the onset of various diseases.In his clinical practice, it has tested these theories on his patients by offering them a nutritional model that classifies hypotoxic. “The power or third medicine” that outlines the principles of this method dietetics, the mechanisms proposed to explain how certain foods may be involved in different pathologies and results Seignalet have observed his patients as a result of nutritional change. These results are sorted by ailments, some ill-treated by conventional medicine, would be put into remission by the treatment applied rigorously. For Seignalet, our genetic heritage from the Paleolithic hunter-gatherers did not have time to adapt to the modern diet. This mismatch is a key to understanding certain diseases. Under the influence of various factors. (genetic enzyme deficiencies, predisposing ground, allergies) and environmental factors such as the modern diet (including, gluten, milk proteins and products of cooking at high temperature) or frequent intake of chemicals such as antibiotics, mucosal Intestinal be attacked, undermined and rendered too porous, allowing easy passage into the bloodstream of macromolecules and bacterial food. According to the theory proposed by Seignalet in this state that is characteristic of hyper-intestinal permeability (leaky gut syndrome), the passage of exogenous molecules cause a chronic inflammatory process and immune response, depending on terrain would lead to the development of autoimmune disease, a disease known for clogging or elimination of diseases known. The food residue or bacteria would be captured by the immune system and then directed to natural excretory, causing inflammation of the target organ. When excess food waste disposal capacity of the body, they accumulate in the extracellular environment, causing fatigue and certain immune system molecules, structures similar to those of the host, penetrate into the cells of a particular organ and alter the operation or jeopardize the long-term survival. Seignalet recommend however to apply this system in substitution for medical treatment. Instead, it involves adopting a lifestyle to prevent disease or improve patient tolerance to treatment. Known for his work in histocompatibility Seignalet published numerous articles on the subject in recognized scientific journals. By no cons did not publish his articles relating to nutrition. On the one hand, it presented no qualitative studies to desired (double blind, and treatment against placebo), other assumptions about the benefits of the system found only hypotoxic little attention in scientific circles. Data on modern hunter-gatherers, following a traditional diet, they suffer little show of some modern diseases (obesity, diabetes type 2 etc.), Regardless of the origin of their main food (meat animal, wild or domestic). Despite the testimony of patients who applied nutrition Seignalet and feel an improvement in their condition, particularly in cases of ankylosing spondylitis, the medical community remains skeptical about the results of this method of nutrition. The National Council of the College of Physicians also emits a warning on this point. Therapeutic efficacy is not recognized by the scientific community, may delay the development of therapeutic and healing can put at stake the lives of patients, illegally practicing medicine.

The book of Dr. Lagacé has already been reviewed by Prof. Ciacci [1] who stressed that “the hypothesis that certain foods, or, better, certain proteins present in the modern diet are linked to chronic diseases is fascinating and deserves significant scientific attention. But evidence-based medicine must now be the reference for any health issue. Scientific research methods require objectivity and control of findings through the randomization of subjects participating in the study”.

From the first chapter dr Lagacè claims “after just ten days I felt more pain in my hands, some sexteen months after the pain disappeared completely even in small fingers worse”, these statements are not, however, supported by any mention of the pathophysiology of chronic pain caused by osteoarthritis. This disease is characterized initially by cartilage degradation, wich often precedes changes in the underlying bone. Patients largely present with pain and disability after significant loss of cartilage has occurred, but it is estimated that up to 40% of individuals with radiological damage have no pain [2]. Recent developments have led to an improved understanding of pain pathways at the molecular level. There is undoubtedly activation of local pain perception phenomena in the most common arthrides including osteoarthritis and rheumatoid arthritis. Several pro-inflammatory mediators may be recruited into the osteoarthritis joint associated with damage, including nerve growth factor (NGF), nitric oxide (NO) and prostanoids [3]. These inflammatory mediators cause localized damage to tissue as well as activating peripheral nociceptor. During chronic disease, the nociceptive system can become sensitized, leading to a heightened sensitivity to noxious stimuli (hyperalgesia), and to pain in response to non-noxious stimuli (allodynia). The activation of these nociceptors is subsequently trasmitted via the dorsal root ganglion, up through the spinothalamic tract to higher cortical centres where signals are processed and perceived as pain.

The CNS is an organ demostrating plasticity in a system that has the capacity to change following peripheral tissue damage. For example, receptor expression may be up- or down-regulated and new synapses may form within thw dorsal horn. Neurones may also alter their threshold of firing. Inflammation may lead to hypersensitivity of peripheral afferent neurones. Activation of such neurons may be influenced not only by localized noxious stimuli associated with inflammation, but structural and biochemical changes also seem to occur in the systems that perceive pain. This theory has been demonstrated by Ivanavicius et al. [4], who showed the variable efficacy of Nonsteroidal Antiinflammatory Drugs over time in a rat model of osteoarthritis with the peak effect at 14 days post-injury. These results give weight to the theory that although inflammation and joint damage cause the initial trigger for pain, sustained exposure to noxious stimuli can cause neuronal plasticity and a subsequent abnormal sensation of pain, unrelated to the inflammation.

Another topic addressed by Dr. lagancè is drug therapy. In this chapter, the doctor says that there are no drugs that can stop the development of chronic inflammatory diseases and that the main goal of medicine is therefore to treat the symptoms of pain through anti-inflammatory and pain medication. In her discussion, however, does not account for innovative therapies that have been developed on the basis of new information on chronic diseases.

In the last years evidence for central pain processing in osteoarthritis provides a rationale for the use of therapeutic agents that target central pain mediation pathways. For example, duloxetine, a selective noradrenaline and serotonin reuptake inhibitor, which is licenzed for the treatment of neuropathic pain, has recently been shown to be effective in the treatment of osteoarthritis knee pain as early as four weeks post treatment [5].The analgesic effect of duloxetine was sustained at 13 weeks and associated with improvement in function. These findings have also been replicated in an Egyptian population of 188 subjects with knee OA, where duloxetine resulted in a statistically significant reduction in pain compared with placebo, with additional improvement in WOMAC scores at 16 weeks [6].

In Conclusion, we believe that chronic pain is, to date, a difficult challenge, but the evidence gathered to date, although limited, show us that we have one more weapon in the treatment of chronic pain associated with rheumatic diseases. Pain is an unpleasant sensory and emotional experience that can not be cured only by the change in lifestyle or alternative diets, the multidisciplinary nature of the same requires the commitment of Anaesthetists Resuscitators - who have taken as the maximum: ‘Pro vitam contra dolorem, semper’ - and other specialists such as a neurologist, radiologist, physiotherapist and psychologist.
